# Prediagnostic concentrations of plasma genistein and prostate cancer risk in 1,605 men with prostate cancer and 1,697 matched control participants in EPIC

**DOI:** 10.1007/s10552-012-9985-y

**Published:** 2012-05-22

**Authors:** Ruth C. Travis, Naomi E. Allen, Paul N. Appleby, Alison Price, Rudolf Kaaks, Jenny Chang-Claude, Heiner Boeing, Krasimira Aleksandrova, Anne Tjønneland, Nina Føns Johnsen, Kim Overvad, J. Ramón Quirós, Carlos A. González, Esther Molina-Montes, Maria José Sánchez, Nerea Larrañaga, José María Huerta Castaño, Eva Ardanaz, Kay-Tee Khaw, Nick Wareham, Antonia Trichopoulou, Tina Karapetyan, Snorri Bjorn Rafnsson, Domenico Palli, Vittorio Krogh, Rosario Tumino, Paolo Vineis, H. Bas Bueno-de-Mesquita, Pär Stattin, Mattias Johansson, Veronika Fedirko, Teresa Norat, Afshan Siddiq, Elio Riboli, Timothy J. Key

**Affiliations:** 1 Cancer Epidemiology Unit, Nuffield Department of Clinical Medicine, University of Oxford, Oxford, OX3 7LF UK; 2 Division of Cancer Epidemiology, German Cancer Research Centre, Heidelberg, Germany; 3 Department of Epidemiology, German Institute of Human Nutrition, Potsdam-Rehbruecke, Nuthetal, Germany; 4 Institute of Cancer Epidemiology, Danish Cancer Society, Copenhagen, Denmark; 5 Department of Cardiology, Center for Cardiovascular Research, Aalborg Hospital, Aarhus University Hospital, Aalborg, Denmark; 6 Department of Epidemiology, School of Public Health, Aarhus University, Aarhus, Denmark; 7 Public Health Directorate, Asturias, Spain; 8 Unit of Nutrition, Environment and Cancer, Cancer Epidemiology Research Programme, Catalan Institute of Oncology, Barcelona, Spain; 9 Andalusian School of Public Health, Granada, Spain; 10 Public Health Department of Gipuzkoa, Basque Government, Donostia-San Sebastian, Spain; 11 CIBER of Epidemiology and Public Health (CIBERESP), Madrid, Spain; 12 Department of Epidemiology, Murcia Regional Health Authority, Murcia, Spain; 13 Public Health Institute of Navarra, Pamplona, Spain; 14 University of Cambridge, Cambridge, UK; 15 MRC Epidemiology Unit, Cambridge, UK; 16 Hellenic Health Foundation, Athens, Greece; 17 WHO Collaborating Center for Food and Nutrition Policies, Department of Hygiene, Epidemiology and Medical Statistics, University of Athens Medical School, Athens, Greece; 18 Molecular and Nutritional Epidemiology Unit, Cancer Research and Prevention Institute–ISPO, Florence, Italy; 19 Nutritional Epidemiology Unit, Fondazione IRCCS Istituto Nazionale dei Tumori, Milan, Italy; 20 Cancer Registry and Histopathology Unit, “Civile–M.P. Arezzo” Hospital, ASP 7, Ragusa, Italy; 21 School of Public Health, Imperial College, London, UK; 22 HuGeF Foundation, Turin, Italy; 23 National Institute for Public Health and the Environment (RIVM), Bilthoven, The Netherlands; 24 Department of Gastroenterology and Hepatology, University Medical Centre Utrecht (UMCU), Utrecht, The Netherlands; 25 Department of Surgery, Urology Service, Memorial Sloan-Kettering Cancer Center, New York, NY USA; 26 Department of Surgical and Perioperative Sciences, Urology and Andrology, Umeå University Hospital, Umeå, Sweden; 27 International Agency for Research on Cancer (IARC), 150 Cours Albert Thomas, Lyon, France; 28 Department of Public Health and Clinical Medicine, Nutritional Research, Umeå University, Umeå, Sweden; 29 Nutritional Epidemiology Group, Nutrition and Metabolism Section, International Agency for Research on Cancer (IARC), 150 Cours Albert Thomas, Lyon, France

**Keywords:** Prospective, Prostate cancer, Plasma, Isoflavone, Genistein, Phyto-estrogen

## Abstract

**Purpose:**

Data from prospective epidemiological studies in Asian populations and from experimental studies in animals and cell lines suggest a possible protective association between dietary isoflavones and the development of prostate cancer. We examined the association between circulating concentrations of genistein and prostate cancer risk in a case–control study nested in the European Prospective Investigation into Cancer and Nutrition.

**Methods:**

Concentrations of the isoflavone genistein were measured in prediagnostic plasma samples for 1,605 prostate cancer cases and 1,697 matched control participants. Relative risks (RRs) for prostate cancer in relation to plasma concentrations of genistein were estimated by conditional logistic regression.

**Results:**

Plasma genistein concentrations were not associated with prostate cancer risk; the multivariate relative risk for men in the highest fifth of genistein compared with men in the lowest fifth was 1.00 (95  % confidence interval: 0.79, 1.27; *p* linear trend = 0.82). There was no evidence of heterogeneity in this association by age at blood collection, country of recruitment, or cancer stage or histological grade.

**Conclusion:**

Plasma genistein concentration was not associated with prostate cancer risk in this large cohort of European men.

## Introduction

Results from several prospective epidemiological studies of dietary isoflavone or soy intake and prostate cancer risk in Asian populations, as well as from experimental studies in animal models and in cell lines, suggest a possible protective association between dietary isoflavones and the development of prostate cancer [1–3]. The contrasting null findings in the majority of observational studies in non-Asian populations have been partly attributed to the typically lower amount of isoflavones consumed in those populations [2]. However, it is difficult to exclude residual confounding by some other aspect of a traditional Asian lifestyle as an explanation for the apparent protective associations observed in Asian populations.

The direct measurement of isoflavones in the circulation, as opposed to dietary isoflavone or soy intake, provides an alternative measure of exposure that captures isoflavone from all sources, including intake that may be inadequately represented in food composition databases [4] and isoflavones that are a product of metabolism by gut microflora [5]. However, published data from large-scale prospective investigations of circulating concentrations of isoflavones in relation to risk are limited and inconsistent [6, 7]. An earlier investigation of plasma phyto-estrogens in 950 men with prostate cancer and 1,042 matched control participants from the European Prospective Investigation into Cancer and Nutrition [8] found a possible inverse association with prediagnostic plasma concentrations of genistein, but no evidence of an association with daidzein, equol, or the lignans enterolactone and enterodiol. Here, we describe the results from an extension of this study, including 1,605 men with incident prostate cancer and 1,697 matched control participants, in which we sought to confirm this association with genistein and to estimate more precisely the risk of prostate cancer, overall and subdivided by tumor characteristics and other factors.

## Materials and methods

Between 1992 and 2000, approximately 500,000 individuals (150,000 men) were recruited into the European Prospective Investigation into Cancer and Nutrition (EPIC) from 23 centers in 10 European countries. The methods of recruitment and study design have been described in detail elsewhere [9]. Participants completed an extensive questionnaire on dietary and non-dietary factors at recruitment, and about 400,000 individuals (of whom 137,000 were men) also provided blood samples. All participants gave written consent for the research, and approval for the study was obtained from the Internal Review Board of the International Agency for Research on Cancer (Lyon, France) and from the local ethics committees in participating countries.

The present study includes prostate cancer cases occurring after blood collection and individually matched male control participants from the eight participating countries that recruited men: Denmark, Germany, Greece, Italy, the Netherlands, Spain, Sweden, and the United Kingdom (UK). Men were eligible for this analysis if they had information available on the date of blood donation and did not have a history of cancer (except non-melanoma skin cancer) at the time of the blood collection.

Follow-up for diagnosis of prostate cancer is provided through record linkage with population-based cancer registries in six of the participating countries: Denmark, Italy, the Netherlands, Spain, Sweden, and the UK. In Germany and Greece, follow-up is active and is achieved through checks of insurance records and cancer and pathology registries as well as via self-reported questionnaires; self-reported incident cancers are verified through medical records. Data on vital status in most EPIC study centers were collected from mortality registries at the regional or national level, in combination with data collected by active follow-up (Greece). The 10th Revision of the International Statistical Classification of Diseases, Injuries and Causes of Death (ICD) was used, and cancer of the prostate was defined as code C61.

We previously reported our findings from measurements in samples from 950 men with prostate cancer (diagnosed between 1999 and 2003) and 1,042 matched control participants that were assayed in 2007 [8]. In the current study, we combined data from those 1992 individuals (designated as phase 1) with 655 additional men who had been subsequently diagnosed with prostate cancer (up to 2006) and their 655 matched control participants (designated phase 2), whose samples were assayed in 2009. Samples for phase 2 were available for EPIC participants from six of the eight countries in phase 1 (phase 2 samples from Denmark and Sweden were not available for the current study).

Case patients were men who were diagnosed with prostate cancer after the date of blood collection and before the end of the study period, defined for each study center by the latest date of follow-up. Laboratory measures for the current analysis were available for a total of 1,605 cases: 288 cases in Denmark, 412 in Germany, 40 in Greece, 146 in Italy, 50 in the Netherlands, 201 in Spain, 94 in Sweden, and 364 in the UK.

Data on the stage and grade of disease at diagnosis were collected from each center, where possible. A total of 1,108 cases (69.0 %) had information on tumor stage; of these, 792 (71.5 %) were classified as localized (tumor [T]-node [N]-metastasis [M] categories T0 or T1 or T2 and N0 or NX and M0, or stage coded in recruitment center as localized), and 316 (28.5 %) were classified as advanced (T3 or T4, N1+, M1, or some combination of these, or stage coded in recruitment center as metastatic). Information on histological grade was available for 1,129 cases (70.3 %); of these, 941 (83.3 %) were classified as low grade (Gleason sum <8 or equivalent, i.e., coded as moderately or as well differentiated) and 188 (16.7 %) were classified as high grade (Gleason sum ≥8 or equivalent, i.e., coded as poorly differentiated or as undifferentiated).

Each case patient was matched to one control participant (with the exception of case patients from Umeå who were matched to two control participants), selected at random among appropriate risk sets consisting of all male cohort members alive and free of cancer (except non-melanoma skin cancer) after the same amount of follow-up time as the index case. An incidence density sampling protocol for control selection was used, such that controls could include participants who became a case later in time, while each control participant could also be sampled more than once. Matching criteria included recruitment center, age at enrollment (±6 months), time of day of blood collection (±1 h), follow-up time (as close as possible), time between blood draw and last consumption of food or drinks (<3, 3–6, >6 h).

### Laboratory assays

Plasma samples for phase 1 and phase 2 were analyzed for genistein in 2007 and 2009, respectively, using ultra performance liquid chromatography/tandem mass spectrometry (UPLC-MS/MS) in the HFL laboratory, Fordham, Cambridgeshire, using an adaptation of a previously published method [10]. The laboratory personnel who conducted the assays were blinded to the case or control status of the participants. Plasma samples from each case–control set were assayed within the same batch and analyzed on the same day.

Three HFL in-house quality control plasma samples were inserted into each assay batch and analyzed in duplicate. These quality control samples contained concentrations of genistein, which reflected low, medium, and high ranges of the calibration chart (concentrations ranging between 0.1 and 500 ng/ml, e.g., equivalent to 0.37–1,850 nmol/l genistein). The average intra-assay and inter-assay coefficients of variation (CVs) for genistein were 3.1 and 4.1 %, respectively, in phase 1 samples, and 3.6 and 7.2 %, respectively, in phase 2 samples. An additional 108 “blinded” quality control samples from a pooled plasma sample (mean genistein concentration, 6.7 ng/ml) were included in phase 2; the average intra- and inter-assay CVs for these samples were 12.3 and 16.0 %, respectively.

The lower limit of quantification, equivalent to the lowest point in the calibration curve, was 0.1 ng/ml; the percentage of samples in which levels were not detectable was 11.8 % overall (15.9 % in phase 1 samples and 5.6 % in phase 2 samples). For individuals with levels below this limit, genistein concentrations were imputed at half the lower limit of detection, that is, 0.05 ng/ml.

### Reproducibility study

To assess the appropriateness of a single measurement of plasma genistein as a marker for long-term exposure to plasma genistein in epidemiological studies, we examined the reproducibility of genistein in plasma samples collected from 100 men and 100 women from a UK-based EPIC-sub-cohort, EPIC-Oxford. We measured circulating concentrations of plasma genistein in two samples from each of the men collected approximately 5 years apart and calculated an intra-class correlation coefficient (ICC) to assess the reliability of the measurements. The intra-class correlation coefficient for plasma genistein was 0.32 (95 % CI 0.14, 0.50).

### Statistical analysis

The concentrations of genistein in cases and controls were compared using a weighted paired *t* test, comparing the value for the case with the value in the matched control participant, or the mean of the values in controls when there were two matched controls in a set [11].

Conditional logistic regression models were applied to calculate the relative risks (odds ratios) for prostate cancer in relation to fifths of genistein concentration using cut points defined by the quintiles among control participants for all centers combined and using the lowest category as reference. Likelihood ratio tests were used to assess heterogeneity, and tests for linear trend were obtained using a continuous variable with values equal to the median concentration within each quintile of plasma genistein concentration.

To examine the effects of potential confounders (other than the matching criteria, controlled for by design), the analyses were repeated including additional variables in the logistic regression models. These variables were smoking (never, past, present), body mass index (BMI, kg/m^2^; in fourths), physical activity (inactive, moderately inactive, moderately active/active) (16), alcohol intake (<8, 8–15, 16–39, ≥40 g/day), marital status (married/cohabiting or not married/cohabiting), and education level (primary or equivalent, secondary, degree level). For each of these variables, a small proportion of values was unknown (<7 % of values missing for each, with the exception of marital status, for which 32 % of values were missing); these values were included in the analyses as a separate category.

Likelihood ratio chi-squared tests were used to examine the heterogeneity of the associations of genistein concentrations with risk of prostate cancer categorized according to prostate tumor stage (localized or advanced), histological grade (low grade or high grade), age at diagnosis (<60 and ≥60 years), and time to diagnosis (less than 4 years after blood collection, four or more years after blood collection). Likelihood ratio chi-squared tests were also used to examine the heterogeneity of the associations of genistein concentrations with prostate cancer risk for participants categorized by age at blood collection (<60 or ≥60 years) and by country of recruitment (8 countries).

Statistical analyses were performed with the Stata 10 statistical software package [12]. The genistein concentrations were logarithmically transformed for statistical analyses to approximately normalize their frequency distribution. All tests of statistical significance were two-sided, and *p* values below 0.05 were considered significant.

## Results

A total of 1,605 men diagnosed with prostate cancer from recruitment until the end of follow-up and 1,697 matched participants without prostate cancer were included in the analyses. Their median age at blood collection was 60 years (range, 43–76 years).

When characteristics of case patients and control participants were compared, the groups did not differ appreciably, but there were some small differences (Table 1). A comparison of the characteristics of cases and controls in phase 1 and phase 2 did not show any clear differences (results not shown).

**Table 1:** Characteristics of prostate cancer patients and control participants

Characteristic	Cases (*n* = 1,605)	Controls (*n* = 1,697)
Age at blood collection, years (SD)	60.2 (6.3)	60.0 (6.2)
Weight, kg (SD)^a^	80.2 (11.3)	80.8 (12.0)
Height, cm (SD)^a^	173.1 (7.0)	173.4 (6.9)
BMI, kg/m^2^ (SD)^a^	26.7 (3.4)	26.9 (3.6)
Smoking, % (*n*)^a^		
Never	32.7 (513)	31.3 (513)
Former	44.1 (693)	41.8 (684)
Current	23.2 (364)	26.9 (440)
Alcohol consumption, % (*n*)^a^		
<8 g/day	34.7 (549)	36.2 (598)
8–15 g/day	20.1 (318)	20.2 (334)
16–39 g/day	25.6 (405)	25.6 (423)
≥40 g/day	19.5 (309)	18.0 (298)
Physical activity, % (*n*)^a^		
Inactive	19.0 (292)	18.0 (286)
Moderately inactive	32.7 (503)	31.5 (500)
Moderately active/active	48.4 (745)	50.5 (801)
Marital status, % (*n*)^a^		
Married or cohabiting	89.3 (971)	89.1 (1,040)
Not married or cohabiting	10.7 (116)	10.9 (127)
Educational attainment, % (*n*)^a^		
Primary or equivalent	37.2 (570)	38.9 (630)
Secondary	36.8 (564)	37.2 (602)
Degree	26.1 (400)	24.0 (388)
Cases only		
Time to diagnosis, % (*n*)^b^		
<2 years	11.5 (184)	–
2–<4 years	18.8 (302)	
4–<6 years	26.5 (426)	
6–<8 years	24.4 (392)	
≥8 years	18.8 (301)	
Year of diagnosis, median (range)	2001 (1994–2006)	–
Age at diagnosis, years (SD)	65.7 (6.3)	–
Stage, % (*n*)		
Localized	49.4 (792)	–
Advanced	19.7 (316)	–
Unknown	31.0 (497)	–
Grade, % (*n*)		
Low grade^c^	58.6 (941)	–
High grade^d^	11.7 (188)	–
Unknown	29.7 (476)	–

Values are means, except where indicated

^a^Unknown for some participants; the calculations of percentages exclude missing values

^b^Time between blood collection and diagnosis among case patients

^c^Gleason score <8 or coded as well or moderately differentiated

^d^Gleason score ≥8 or coded as poorly differentiated or undifferentiated

Men with prostate cancer were diagnosed an average 5.5 years after blood collection (range <1–15.1 years), and the median age at diagnosis was 65 years (range, 44–85 years).

Table 2 shows the distribution of plasma concentrations of genistein in controls in the eight countries participating in the study. There was substantial variation in median concentrations between the countries, with participants in the UK and the Netherlands generally having the highest concentrations of genistein and participants in Greece having the lowest concentration.

**Table 2:** Median (5th–95th percentile) concentrations of genistein^a^ among controls by country and study phase

Genistein (ng/ml)	Denmark	Germany	Greece	Italy	Netherlands	Spain	Sweden	UK	Total
Phase 1									
Number	288	201	9	62	25	93	186	178	1,042
Median (5–95 percentile)	2.0 (0.05–23.8)	2.2 (0.05–23.5)	0.05 (0.05–17.2)	1.3 (0.05–10.3)	4.6 (0.2–40.1)	1.2 (0.05–7.4)	1.1 (0.05–14.9)	5.4 (0.2–41.5)	2.1 (0.05–24.5)
Phase 2									
Number	0	211	31	84	25	108	0	196	655
Median (5–95 percentile)	–	1.6 (0.1–13.7)	0.3 (0.05–5.0)	1.1 (0.05–5.7)	6.5 (1.5–26.2)	1.0 (0.05–4.9)	–	3.1 (0.1–24.0)	1.7 (0.05–16.1)
Total									
Number	288	412	40	146	50	201	186	374	1,697
Median (5–95 percentile)	2.0 (0.05–23.8)	1.9 (0.05–21)	0.3 (0.05–5.05)	1.15 (0.05–6.9)	5.2 (0.3–27.9)	1.2 (0.05–5.4)	1.1 (0.05–14.9)	4.3 (0.2–30.1)	1.9 (0.05–21.6)

^a^For participants with undetectable genistein levels, data were imputed at 0.05 ng/ml, half the lower limit of detection

When the distribution of plasma concentrations of genistein in case patients and control participants were compared, the differences between concentrations in cases and controls were small in both phases (a difference of 0.3 ng/ml in both phases), and overall, there was no significant difference in genistein concentrations between cases and controls (median concentration was 1.9 ng/ml in both cases and controls, *p* = 0.20).

We examined the relative risks (RR) for prostate cancer by plasma genistein concentration in phase 1 and in the new samples from the 655 men with prostate cancer and 655 controls (phase 2), and in a combined sample of 1,605 cases and 1,697 controls (phases 1 and 2). In contrast to our earlier study (phase 1), in phase 2 we observed a statistically significant positive association between risk of prostate cancer and concentration of genistein, with or without adjustment for potential confounders (Table 3): compared with men in the lowest fifth of genistein concentration, men in the highest fifth had a RR of disease of 1.57 (95 % CI: 1.05, 2.33; *p* linear trend = 0.02). However, when data from phase 1 and phase 2 were combined, there was no significant association: compared with the men in the lowest fifth of genistein concentration, men in the highest fifth had a RR of disease of 0.98 (95 % CI: 0.77, 1.23; *p* linear trend = 0.95). Results were similar when the analysis was additionally adjusted for smoking, BMI, physical activity, alcohol intake, marital status, and education level. There was no statistical evidence of heterogeneity in the trends in risk of prostate cancer according to age at blood collection, body mass index, or country of recruitment. In addition, there was no evidence of heterogeneity in the associations of genistein concentrations with risk of prostate cancer categorized according to prostate tumor stage, histological grade, age at diagnosis, or time to diagnosis (Table 3).

**Table 3:** Multivariable-adjusted relative risks (95 % CI)^a^ for prostate cancer by fifth of plasma genistein concentration, subdivided by selected factors

	Fifth of genistein concentration (ng/ml)					*p* for trend^c^	*p* for heterogeneity of trends^d^
	1 (0.05–0.30)	2 (0.40–1.30)	3 (1.40–2.60)	4 (2.70–6.00)	5 (6.10–567.70)		
Overall							
Cases/controls (*n*)	338/357	326/333	282/329	326/344	333/334		
RR (95 % CI)	1 (reference)	1.01 (0.81–1.26)	0.86 (0.68–1.09)	0.94 (0.74–1.19)	0.98 (0.77–1.23)	0.95	
Adjusted RR (95 % CI)^b^	1 (reference)	1.03 (0.82–1.29)	0.88 (0.69–1.12)	0.95 (0.75–1.21)	1.00 (0.79–1.27)	0.82	
Study phase							
Phase 1							
Cases/controls (*n*)	217/219	205/209	171/201	185/205	172/208		
Adjusted RR (95 % CI)^b^	1 (reference)	1.00 (0.76–1.34)	0.81 (0.60–1.10)	0.80 (0.59–1.08)	0.74 (0.54–1.00)	0.05	<0.0001
Phase 2							
Cases/controls (*n*)	121/138	124/126	118/129	137/135	155/127		
Adjusted RR (95 % CI)^b^	1 (reference)	1.20 (0.81–1.76)	1.12 (0.75–1.67)	1.26 (0.84–1.87)	1.57 (1.05–2.34)	0.03	
Stage of disease							
Localized stage							
Cases/controls (*n*)	182/199	166/177	130/161	156/186	158/141		
Adjusted RR (95 % CI)^b^	1 (reference)	1.03 (0.76–1.41)	0.84 (0.60–1.19)	0.86 (0.62–1.20)	1.13 (0.81–1.57)	0.32	0.22
Advanced stage							
Cases/controls (*n*)	67/71	61/56	57/61	66/67	65/73		
Adjusted RR (95 % CI)^b^	1 (reference)	1.20 (0.69–2.09)	0.98 (0.56–1.72)	0.96 (0.53–1.74)	0.88 (0.50–1.55)	0.42	
Grade of disease							
Low grade							
Cases/controls (*n*)	219/214	189/208	148/192	193/206	192/204		
Adjusted RR (95 % CI)^b^	1 (reference)	0.84 (0.62–1.13)	0.67 (0.49–0.93)	0.79 (0.57–1.09)	0.79 (0.57–1.08)	0.50	0.26
High grade							
Cases/controls (*n*)	37/47	32/28	37/40	41/44	41/35		
Adjusted RR (95 % CI)^b^	1 (reference)	1.45 (0.66–3.18)	1.28 (0.61–2.70)	1.04 (0.49–2.21)	1.57 (0.72–3.40)	0.36	
Age at blood collection							
<60 years							
Cases/controls (*n*)	179/178	166/168	130/168	150/132	135/151		
Adjusted RR (95 % CI)^b^	1 (reference)	0.99 (0.73–1.36)	0.74 (0.53–1.06)	1.14 (0.80–1.63)	0.87 (0.61–1.24)	0.57	0.49
≥60 years							
Cases/controls (*n*)	150/151	154/153	147/155	174/202	194/176		
Adjusted RR (95 % CI)^b^	1 (reference)	1.00 (0.70–1.41)	0.94 (0.67–1.34)	0.82 (0.58–1.15)	1.10 (0.78–1.55)	0.36	
Age at diagnosis							
<60 years							
Cases/controls (*n*)	72/72	51/59	54/54	53/46	47/54		
Adjusted RR (95 % CI)^b^	1 (reference)	0.95 (0.54–1.67)	1.01 (0.54–1.86)	1.20 (0.66–2.19)	0.74 (0.39–1.39)	0.35	0.80
≥60 years							
Cases/controls (*n*)	266/285	275/274	228/275	273/298	286/280		
Adjusted RR (95 % CI)^b^	1 (reference)	1.05 (0.82–1.34)	0.85 (0.65–1.10)	0.91 (0.70–1.18)	1.02 (0.79–1.33)	0.66	
Time between blood collection and diagnosis							
<48 months							
Cases/controls (*n*)	100/104	80/94	92/95	94/78	110/112		
Adjusted RR (95 % CI)^b^	1 (reference)	0.96 (0.62–1.48)	1.03 (0.66–1.60)	1.25 (0.79–1.97)	1.02 (0.66–1.56)	0.97	0.86
≥48 months							
Cases/controls (*n*)	238/253	246/239	190/234	232/266	223/222		
Adjusted RR (95 % CI)^b^	1 (reference)	1.08 (0.82–1.41)	0.82 (0.62–1.10)	0.85 (0.64–1.14)	0.98 (0.73–1.31)	0.97	

^a^Case patients and control participants were matched on recruitment center, age at enrollment (±6 months), time of day of blood collection (±1 h), follow-up time (as close as possible), time between blood draw, and last consumption of food or drinks (<3, 3–6, >6 h)

^b^Adjustment was made for smoking (never, past, present), physical activity (inactive, moderately inactive, active), alcohol intake (<8, 8–15, 16–39, ≥40 g/day), marital status (married or cohabiting, not married or cohabiting), education (primary or none, secondary, degree level), and BMI (fourths)

^c^Test for trend obtained by replacing the categorical variable with a continuous variable equal to the median concentration within each fifth of plasma genistein concentration

^d^Test for heterogeneity between the trends obtained by replacing the categorical variable with a continuous variable equal to the median concentration within each fifth of plasma genistein concentration

We conducted three exploratory analyses in which we defined a baseline reference group as men with plasma genistein less than 5 ng/ml (1,205 cases) and the highest exposure groups with cut points of 20 ng/ml (83 cases), 30 ng/ml (43 cases), and 50 ng/ml (23 cases), respectively. After adjustment for potential confounders, compared with men in the baseline group (<5 ng/ml), men in the highest groups (genistein concentrations ≥20, ≥30 and ≥50 ng/ml, respectively) had adjusted RRs for prostate cancer of 0.91 (0.67–1.24), 0.83 (0.54–1.27), and 0.96 (0.54–1.73) (data not shown).

## Discussion

In this large European prospective study, plasma concentration of genistein was not associated with risk of prostate cancer. Furthermore, there was no evidence for heterogeneity in the association by tumor stage or grade or other factors. Strengths of the current analysis include the large sample size and the varied dietary habits of the study population, with the associated variation in plasma genistein levels across the different countries participating in the study (a 14-fold difference in concentration between Greece and the Netherlands, the countries with the lowest and highest levels, respectively) [13]. We are unable to identify any material differences between participants in phase 1 and phase 2 of our study besides time between blood collection and diagnosis, age at diagnosis, and country of recruitment (samples from Denmark and from Umea in Sweden were not available for assaying in phase 2). Given the lack of heterogeneity in findings by these factors and the relatively small sample sizes in each phase, the marginally significant but opposing associations we observed in the two phases were most likely due to chance.

The null findings from this current study are consistent with findings from two smaller Japanese nested case–control studies, which included 40 and 201 men with prostate cancer, respectively [6, 7]. In the UK, results from the EPIC-Norfolk cohort, including 191 cases [14], the majority of whom are included in the current study, also showed no significant associations between serum concentrations of genistein and risk of prostate cancer. Our results are also consistent with the findings from two recent meta-analyses of dietary isoflavone intake in relation to risk, both of which found no evidence of a significant association in non-Asian populations [1, 2]. Thus, our data provide little support for a biologically relevant effect of genistein as manifested in an association with prostate cancer risk at a range of dietary exposures typical of Western populations. There has been much discussion about the levels of dietary phyto-estrogens needed to exert a biological effect, and it may be that despite the oversampling of vegetarians and vegans in a British sub-cohort of EPIC (EPIC-Oxford), levels of exposure are too low to be biologically relevant [15]. The median circulating genistein concentration in the top fifth of the distribution in this study, 14 ng/ml, is approximately seven times lower than the average concentration of 99 ng/ml found in a study in Japanese men [6]. In order to assess possible associations with higher concentrations of plasma genistein, we conducted three exploratory analyses, comparing risk among men with plasma genistein greater or equal to 20, 30, and 50 ng/ml, respectively, with risk among a baseline group with genistein concentrations less than <5 ng/ml. These cut points yielded 83, 42, and 23 cases in the highest exposure categories, respectively, and relative risks in these top categories compared with the baseline group of 0.91 (0.67–1.24), 0.83 (0.54–1.27), and 0.96 (0.54–1.73). Thus, even with higher plasma concentrations of genistein, there is no evidence for an association, although of course there are very small numbers with high levels of exposure reflecting the generally low intake of isoflavones in European populations.

Screening for prostate cancer with prostate-specific antigen is not routinely performed in any of the EPIC countries, although testing for prostate cancer by using the serum concentration of prostate-specific antigen (PSA) has become more widely used during the follow-up period of the present study. Data on PSA use in the EPIC cohort are not available, but studies of annual rates of PSA testing in older middle-aged men in some of the participating countries suggest rates of 6 % in England and Wales, 7 % in Netherlands, 9 % in Spain, and 16 % in Italy, compared with approximately 38 % in US whites [16–20]. In the current study, we had data on stage for 69 % of the cases, of which 29 % (*n* = 316) were advanced; this is a higher proportion than that in recent North American studies, but it provided only a moderate sample size. When we investigated the relationship of plasma genistein with prostate cancer risk subdivided by selected characteristics, including tumor subtype, we found no evidence that the relationship between plasma genistein and prostate cancer risk differed according to the characteristics of the tumors or the participants, including stage or histological grade of the disease and age at blood collection. These results contrast with those from the case–control study nested within the Japan Public Health Center-based Prospective Cohort Study, which found a significant inverse association between circulating isoflavones and risk of localized prostate cancer but not advanced stage disease [7]. However, the small numbers of participants in the subgroups in this Japanese study (144 and 48 men with localized and advanced stage disease, respectively) mean these findings should be interpreted cautiously.

A limitation of this study is the use of a single measurement of circulating genistein, rather than multiple measurements, as a marker of isoflavone exposure. While previous studies have shown that serum isoflavones are good markers of dietary isoflavone intake [21], a single measurement of circulating genistein in a Western population with episodic and relatively low intake of isoflavones may not be a very reliable indicator of long-term exposure [22, 23]. Findings from two studies have suggested that a single measure of circulating genistein may not provide a good index of long-term exposure in populations from the USA (ICCs of 0.22 and 0.28, respectively, for genistein measured in serum samples taken 1–2 years apart) [22, 23]. Results from our reliability study in the EPIC-Oxford sub-cohort are similar (ICC = 0.32). Thus, measurement error due to the use of a single measurement of circulating genistein concentration in our study may be masking any association that exists with prostate cancer risk.

In conclusion, the findings from this large prospective study provide no evidence for an association between concentrations of circulating genistein and risk of prostate cancer in European men. The data presented illustrate the challenges of studying the association between circulating isoflavones and cancer risk in Western studies, in which intake of dietary isoflavones is usually infrequent and relatively low.

## Abbreviations

CI: Confidence interval
EPIC: European Prospective Investigation into Cancer and Nutrition
RR: Relative risk
